# Community-Based Settings and Sampling Strategies: Implications for Reducing Racial Health Disparities Among Black Men, New York City, 2010–2013

**DOI:** 10.5888/pcd11.140083

**Published:** 2014-06-19

**Authors:** Helen Cole, Joseph Ravenell, Antoinette Schoenthaler, R. Scott Braithwaite, Joseph Ladapo, Sherry Mentor, Jennifer Uyei, Chau Trinh-Shevrin

**Affiliations:** Author Affiliations: Joseph Ravenell, Antoinette Schoenthaler, R. Scott Braithwaite, Joseph Ladapo, Sherry Mentor, Jennifer Uyei, Chau Trinh-Shevrin, New York University School of Medicine, New York, New York.

## Abstract

**Introduction:**

Rates of screening colonoscopies, an effective method of preventing colorectal cancer, have increased in New York City over the past decade, and racial disparities in screening have declined. However, vulnerable subsets of the population may not be reached by traditional surveillance and intervention efforts to improve colorectal cancer screening rates.

**Methods:**

We compared rates of screening colonoscopies among black men aged 50 or older from a citywide random-digit–dial sample and a location-based sample focused on hard-to-reach populations to evaluate the representativeness of the random-digit–dial sample. The location-based sample (N = 5,568) was recruited from 2010 through 2013 from community-based organizations in New York City. Descriptive statistics were used to compare these data with data for all black men aged 50 or older from the 2011 cohort of the Community Health Survey (weighted, N = 334) and to compare rates by community-based setting.

**Results:**

Significant differences in screening colonoscopy history were observed between the location-based and random-digit–dial samples (49.1% vs 62.8%, *P* < .001). We observed significant differences between participants with and without a working telephone among the location-based sample and between community-based settings.

**Conclusions:**

Vulnerable subsets of the population such as those with inconsistent telephone access are excluded from random-digit–dial samples. Practitioners and researchers should consider the target population of proposed interventions to address disparities, and whether the type of setting reaches those most in need of services.

## Introduction

Disparities in the incidence of and mortality from chronic diseases such as colorectal cancer (CRC) contribute to a lower life expectancy for black men (mean age, 71.8 y) compared with white men (mean age, 76.5 y) in the United States (1–3). In New York City (NYC) in 2003, vital statistics indicated significant racial disparities in mortality from CRC. Meanwhile, disparate rates of timely screening colonoscopy by race were noted among participants in the Community Health Survey (CHS), an annual random-digit–dial (RDD) survey of approximately 10,000 NYC residents; the lowest screening rates were found among racial groups with the highest CRC-related mortality (4). As a result, the NYC Department of Health and Mental Hygiene (DOHMH) instituted a multifaceted program to increase CRC screening rates and reduce disparities (4). By 2007, the CHS indicated reduced racial disparities and substantial improvements in CRC screening rates among all older New Yorkers, prompting the NYC Citywide Colon Cancer Control Coalition to declare that “powerfully, racial and ethnic colon cancer screening disparities among blacks, whites, Hispanics and Asians have been eliminated” (4,5). Although this achievement was remarkable as a population-wide health strategy, racial disparities in CRC mortality persisted over the same 5-year period. Therefore, it is unclear whether CRC screening rates improved across all subgroups, particularly in communities that may be excluded by RDD sampling such as people from vulnerable subsets of the population (ie, those without consistent telephone access), potentially obscuring disparities within subgroups.

We tested this hypothesis by examining 2 sampling methods used to capture population-level data for older black men: a weighted RDD sample and a location-based community sample. Among the location-based sample, we compared differences in self-reported history of CRC screening by 1) people with access to a working phone and 2) the type of community setting.

## Methods

### Study design and data collection

We collected screening data between 2010 and 2013 as part of the recruitment for the Men’s Health Initiative (MHI), consisting of 2 community-based randomized controlled trials testing behavioral interventions to improve blood pressure control and encourage CRC screening among black men aged 50 or older in NYC. This study is based on a cross-sectional analysis of the screening data for all screened participants, regardless of their eligibility for the parent trials. The New York University School of Medicine Institutional Review Board approved the study, and all participants provided verbal informed consent.

For comparison, we used the NYC DOHMH CHS 2011 public use data set (6). The CHS is modeled after the Behavioral Risk Factor Surveillance System, surveying approximately 10,000 NYC adults annually. The sample is weighted on the basis of probability of being selected (number of adults in the household and number of telephone lines) and a poststratification adjustment to the population for each United Hospital Fund area based on age, sex, race/ethnicity, telephone usage category (landline only, dual use, or cellular telephone only), marital status, education, and number of people in a household (7). For this study, analyses were limited to the weighted sample of 334 black men aged 50 or older included in the 2011 CHS data set. The cooperation rate for the 2011 CHS (number of participants divided by the number of people in the sample who were contacted and deemed eligible) was 89.1% (6).

### MHI settings and participants

Self-reported sociodemographic data and history of CRC screening were obtained from the MHI sample of 5,636 black men aged 50 or older. Participants were intercepted in NYC neighborhood venues, including barbershops, churches, soup kitchens, mosques, senior centers, health fairs, and social service agencies. Neighborhoods with large populations of older black men were identified through 2010 Census data and the DOHMH. Sites were identified through referral, by using Internet searches, and by neighborhood walking tours by study staff. Study staff visited each venue, explained the study, and asked if the venue would be interested in participating as a study site. At each site, the leaders (eg, church leaders, barbershop owners) were consulted to identify the best time to conduct recruitment events. Sites that provided ongoing services (eg, soup kitchens, social service agencies, barbershops) were visited on multiple days to ensure that all interested participants in the target demographic had been screened. At the planned recruitment events, study staff provided blood pressure screening to all adult men and women in the community who wished to be screened. All men who fit the inclusion criteria of 1) self-identifying as black, 2) being aged 50 or older, and 3) being proficient in English were invited to participate in the eligibility screening.

### Variables

In the MHI data, 2 items were used to determine history of colonoscopy: 1) a dichotomous screening history question and 2) for those who had been screened, a question about type of last screening test. We used a standard self-report item to assess self-rated general health. Two items assessed demographics: level of education and access to a working telephone (as an indicator of socioeconomic status). Due to the protection of participant privacy, no identifiable information was collected on the screening questionnaire, limiting the availability of data to these 2 socioeconomic indicators.

We classified settings into the following categories: churches, mosques, barbershops, senior centers, social service locations, and health fairs. Churches included people who were likely to be members of church congregations, as recruitment events at these locations occurred before or after church services or before or after meetings of men’s ministries or health ministries attended primarily by church congregants. Social services included soup kitchens, food pantries, and organizations providing other types of services such as job counseling or case management. Although some of these organizations were free-standing secular institutions, many were run by churches or other faith-based organizations. Similarly, health fairs included those conducted by churches that targeted the church’s surrounding neighborhoods and not only the church congregation; these events were generally held outdoors on a day when church services or meetings were not in session. We also included health fairs conducted by secular community organizations. Participants recruited at barbershops included not only barbershop customers, but also potential participants in the neighborhood surrounding the barbershop. We recruited at mosques after Jumm’ah prayer when most congregants were present. We visited senior centers during weekdays. We excluded people recruited at community-based organizations such as fraternities or community board meetings (N = 38) due to the small sample size, resulting in a final sample size of 5,589.

### Data analysis

We conducted analyses using SPSS version 20 (SPSS Inc, Cary, North Carolina). We compared sociodemographic data, general health data, and CRC screening history among black men aged 50 or older from the CHS 2011 RDD sample to the MHI participants using χ^2^ tests. The same or similar items were matched across the 2 data sources. We then compared participants from the MHI data set with no working telephone to those with a working telephone. The latter group was also compared with the RDD sample (inclusion in RDD samples relies on having a working telephone). Finally, we compared participants across MHI community settings. For all analyses, *P* < .05 was considered significant.

## Results

All participants were black men aged 50 or older. Compared with the CHS sample, the MHI sample had lower educational attainment, worse self-reported health, and a lower rate of CRC screening (49% vs 63%, *P* < .001) (Table 1). Ten percent of the MHI sample had no working telephone. Participants without a working telephone had lower educational attainment, worse self-reported health, and lower CRC screening rates (Table 2). After excluding MHI participants with no working telephone, the CHS sample still had higher rates of screening colonoscopies (62.8% vs 49.3%, *P* < .001).

**Table 1:** Demographic and Health Characteristics of Black Men Aged 50 or Older, Community Health Survey (CHS) and Men’s Health Initiative (MHI), New York City, 2010–2013

Characteristic	CHS 2011 RDD Survey (N = 334), Weighted %^a^	MHI Community-Based Survey (N = 5,636), %^a^	*P* Value^b^
**Highest grade or year of school**			
Less than high school (through 11th grade)	24.5	32.0	<.001
GED or high school graduate	28.3	36.3	
Some college or higher	47.2	31.7	
**Has a working telephone at the time of survey**	100	90.5	<.001
**Self-rated general health**			
Excellent, very good, or good	76.1	68.6	<.001
Fair or poor	23.9	31.5	
**Ever had a colonoscopy**	62.8	49.1	<.001

Abbreviations: RDD, random digit–dial; GED, general educational development.

a Percentages weighted to population totals based on sex, age, race/ethnicity, marital status, education and the number adults in the household (7).

b χ^2^ test used to determine *P* values.

**Table 2:** Demographic and Health Characteristics of Black Men Aged 50 or Older (N = 5,568),^a^ by Telephone Access Status, Men’s Health Initiative, New York City, 2010–2013

Item	Working Telephone (N = 4,829), %	No Working Telephone (N = 508), %	*P* Value^b^
**Highest grade or year of school**			
Less than high school (through 11th grade)	30.3	46.5	<.001
GED or high school graduate	37.0	31.1	
Some college or higher	32.8	22.4	
**Self-reported general health**			
Excellent, very good, or good	68.8	63.0	.003
Fair or poor	31.2	37.0	
**Ever had a colonoscopy**	49.2	41.8	.001

Abbreviation: GED, general educational development.

a Values do not sum to total value for N due to missing data.

b χ^2^ test used to determine *P* values.

MHI participants from churches had the highest educational attainment compared with participants from other settings; 55.4% of church participants had at least some college education (Table 3). Only 3.5% of participants at churches lacked access to a working telephone compared with 12.5% of participants recruited from social service agencies (*P* < .001). Results also varied by setting type with regard to self-reported general health; only 18.2% of participants from churches reported fair or poor health compared with 35.1% of participants from senior centers (*P* < .001). Finally, 72.7% of senior center participants and 71.9% of church participants reported having ever had a screening colonoscopy compared with 47.1% of participants from social service locations, 55.0% of health fair participants, 46.1% of barbershop participants, and 30.6% of mosque participants (*P* < .001).

**Table 3:** Demographics, Self-Rated Health, and Colorectal Cancer Screening Status Among Black Men Aged 50 or Older (N = 5,589), by type of Recruitment Site, Men’s Health Initiative, New York City, 2010–2013

Item	Type of Recruitment Site						*P* Value^a^
	Churches (N = 305)	Social Services (N = 2,066)	Health Fairs (N = 578)	Barbershops (N = 2,370)	Senior Centers (N = 132)	Mosques (N = 138)	
	%						
**Highest grade or year of school**							
Less than HS (through 11th grade)	12.9	36.6	29.4	30.8	27.9	42.6	<.001
GED or HS graduate	31.7	36.0	34.4	38.4	37.2	27.2	
Some college or higher	55.4	27.4	36.2	30.8	34.9	30.1	
**Has a working telephone at the time of survey**	96.5	87.5	91.1	91.8	92.9	95.3	<.001
**Self-rated general health**							
Excellent, very good, or good	81.8	67.2	71.4	66.8	64.9	76.3	<.001
Fair or poor	18.2	32.8	28.6	33.2	35.1	23.7	
**Ever had a colonoscopy**	71.9	47.1	55.0	46.1	72.7	30.6	<.001

Abbreviations: GED, general educational development; HS, high school.

a χ^2^ test used to determine *P* values.

## Discussion

To decrease racial disparities in health, population-level interventions must reach those who are most in need. Likewise, accurate documentation of progress in reducing health disparities relies on the inclusion of diverse populations, including vulnerable subgroups, in surveillance efforts. We found considerable differences between location-based and RDD samples of older black men in NYC in terms of education and self-reported health, with the most striking difference being for CRC screening. Our data indicate that surveillance data must include methods for reaching people who may be more vulnerable than those reached in RDD samples to sufficiently capture disparities. Moreover, community-based interventions should include varied settings rather than concentrating efforts in 1 location to ensure reaching those who are most in need.

Despite underrepresentation in research, studies indicate that black men in the United States experience worse health outcomes than any other racial/ethnic or gender group (8–11). The institutionalization of racism and structural inequalities have created lasting health and socioeconomic inequalities affecting blacks in the United States, and black men specifically face structural disadvantages that undermine their likelihood of being included in surveillance efforts and health promotion programs (12–14). For example, black men are more likely than others to be imprisoned, and prisoners are excluded from most surveillance sampling frames (14–16). As many as 1 in 3 black men will be imprisoned at some point in their lives (16). Black men are also more likely to be unemployed and to have unstable housing or experience homelessness (15). Therefore, black men are less likely than others to be represented in RDD samples, other household-based samples, or community-based recruitment. In medical settings, black men may not be reached for intervention or surveillance purposes, because they are less likely than others to receive regular health care or to have a primary care provider (15). In addition, mistrust of research or medicine by blacks may result in the active avoidance of health-related programs and research in any setting (17). Furthermore, those reached using various techniques may be segmented, with no one setting or sampling strategy being truly representative of the population.

Our findings indicated important differences between people with and without working telephones, suggesting a potential for noncoverage bias in RDD population estimates of CRC screening. Also of note, 9.5% of our location-based sample had no working telephone, which is almost twice the national estimate of households with no telephone (18). Among the church-based sample, only 3.5% lacked a working telephone, indicating that these people would be more likely to be sampled using an RDD approach.

Although random sampling strategies are generally considered more representative of the general population, few studies have empirically examined the representativeness of these samples, perhaps due to a lack of appropriate comparison groups. One study found that location intercept-sampling, similar to our approach, resulted in a sample that had greater connection with their community, resulting in potential selection bias when compared with household-based sampling (19). However, this finding may have been due to the types of locations included. Our sample included a diverse selection of settings, which we believe attracted men from diverse socioeconomic backgrounds, regardless of their engagement with other community activities or the health care system. This inclusion also enabled us to examine the potential differences between samples intercepted in different settings. Understanding the discrepancy in CRC screening by setting and sampling technique has public health implications, as black men have higher CRC incidence and mortality than do other populations in the United States (3). In NYC, citywide RDD sampling indicates that disparities in screening by race have been dramatically reduced, which should lead to reductions in disparities in incidence and mortality (4). However, this apparent progress may reflect noncoverage bias and the omission of more disadvantaged segments of the population.

In recent years, churches have become a popular venue for implementing health programs, including interventions to promote cancer screening (20–22). Past research on interventions conducted in religious organizations point to the social structure of such organizations, such as the innate social support system that may benefit congregants’ health, and available resources for health programming (21,23). Thus, it is not surprising that our data indicate that black men intercepted at churches were significantly more likely than others to report prior CRC screening. Among our participants, 72% of men from churches had a prior screening colonoscopy, which was greater than the 63% RDD estimated screening rate for black men in NYC. Faith-based recruitment and interventions remain important vehicles for health promotion in black communities, but consideration should extend to community sites that serve marginalized segments of this population to ultimately eliminate racial disparities in CRC screening, morbidity, and mortality. Our data indicate there may be a need to broaden the reach of interventions beyond church congregations to include the surrounding community. As more affluent congregants have moved from the inner city to the suburbs, many black churches have remained in their original locations, in part due to a commitment to social justice and serving those left behind through social services and outreach (24). Partnerships with church-based social services such as food pantries and soup kitchens offer a viable way to reach those most in need, and such interventions would also reduce disparities. Participants at senior centers were also more likely than other participants to have had a colonoscopy. However, senior center attendees are older on average (mean age, 76 y) than the older adult population of NYC (25). Thus, senior center attendees may be more likely than others to qualify for Medicare and to have had more time to meet CRC screening recommendations. Similar to those of churches, the core functions of senior centers include providing social engagement, links to resources and services, and promoting health (25).

Participants from mosques, social services, and barbershops exhibited CRC screening rates that were far below the citywide RDD estimates for black men. Participating mosques were largely those serving the African immigrant community, congregants of which may lack access to services due to socioeconomic and immigration status, and availability of culturally appropriate care may contribute to the low screening rates among these participants (26). Despite their potential to reach many immigrant communities, mosques are included in few studies as settings for health promotion programs (26).

Conversely, many barbershop-based interventions target health issues similar to those targeted by church-based interventions. Because barbershops are important community centers for the black community, many men spend time at and around barbershops in their neighborhoods even when they are not getting their hair cut (27,28). Moreover, although we did not collect information on place of residence, we observed that many of the men recruited from barbershops were attracted from the surrounding neighborhood. This finding is congruent with Wright and Calhoun’s findings that barbershops are locations where men from the surrounding community are able to spend time together and escape the solitude of their homes (29). Thus, barbershops have salience for interventions targeting hard-to-reach populations such as black men.

### Study strengths and limitations

This study included only black men aged 50 or older, so results may not be generalizable to women, younger men, or people of other races. This study took place in NYC, which is different in many ways from other US cities. For example, we observed that barbershops in neighborhoods with high volumes of foot traffic tended to yield more study participants. In cities or neighborhoods that rely more heavily on cars for transportation, our findings may not be as applicable. Although people may travel to inner-city churches from more affluent suburban areas, this may also be true for suburbanites seeking black-owned barbershops (24). Although barbershops may attract more local residents than do churches, we were not able to determine whether this is true for our sample, because we did not collect information on place of residence.

We used location-based convenience sampling, which may also limit the generalizability of the results. The potential for multiplicity may have biased the results due to the sampling techniques. However, no incentive was provided for participating in the survey, and a small study staff attended events at each location, maximizing the possibility that they would be familiar with potential repeat participants. As data were collected for the purpose of eligibility screening for 2 randomized controlled trials, only items relevant to eligibility for these trials were included. Thus, few data were available on demographics, insurance status, or health care access, which would have provided insight into the reasons for the observed differences between recruitment settings and sampling types. However, the small amount of time and effort required to complete our survey allowed us to sample a large group of older black men from many different settings.

### Conclusions

Racial disparities in health and health care persist, improving little over the past 10 years (30). Interventions to address racial and socioeconomic disparities are important to improve the health of black men, who are both underrepresented in research and have the lowest life expectancy in the United States, in part due to increased prevalence of preventable and treatable chronic diseases (1,8). To reach minorities, particularly blacks and Hispanics, interventions have often targeted churches, barbershops, and other community settings. Our results show that RDD sampling may not adequately characterize health-related disparities. Surveillance efforts and subsequent health promotion interventions to decrease disparities in health should include varied settings with a focus on men that frequent social services and who may be reached through barbershops or settings such as mosques where immigrant communities may be reached. Practitioners and researchers should carefully consider the target population of proposed interventions to address disparities and whether the reach and type of recruitment setting is aligned with the respective populations.
