# Erratum, Vol. 11, June 19 Release

**DOI:** 10.5888/pcd11.140083e

**Published:** 2014-07-10

In the article “Community-Based Settings and Sampling Strategies: Implications for Reducing Racial Health Disparities Among Black Men, New York City, 2010–2013,” an author, Joseph Ravenell, MD, MS, was omitted from the byline as a result of an editorial error. The correction was made to our website on July 1, 2014, and appears online at http://www.cdc.gov/pcd/issues/2014/14_0083.htm. We regret any confusion or inconvenience this error may have caused.

